# Supplementary material for: A New Indicator to Differentiate Thyroid Follicular Inclusions in Cervical Lymph Nodes from Patients with Thyroid Cancer
Source: Int J Mol Sci. 2022 Dec 28;24(1):490. doi: 10.3390/ijms24010490 (PMC9820803; doi:10.3390/ijms24010490)
Supplement: Supplementary file 1 [file ijms-24-00490-s001.zip › ijms-2006515-supplementary.pdf]

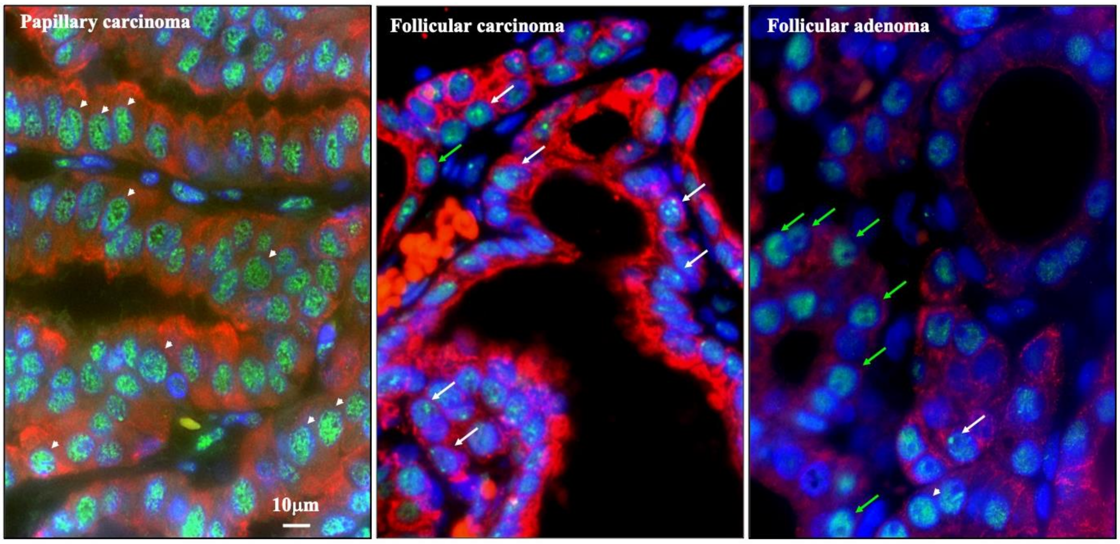

Figure S1. Immunofluorescence for 53BP1 expression in Figure 1.

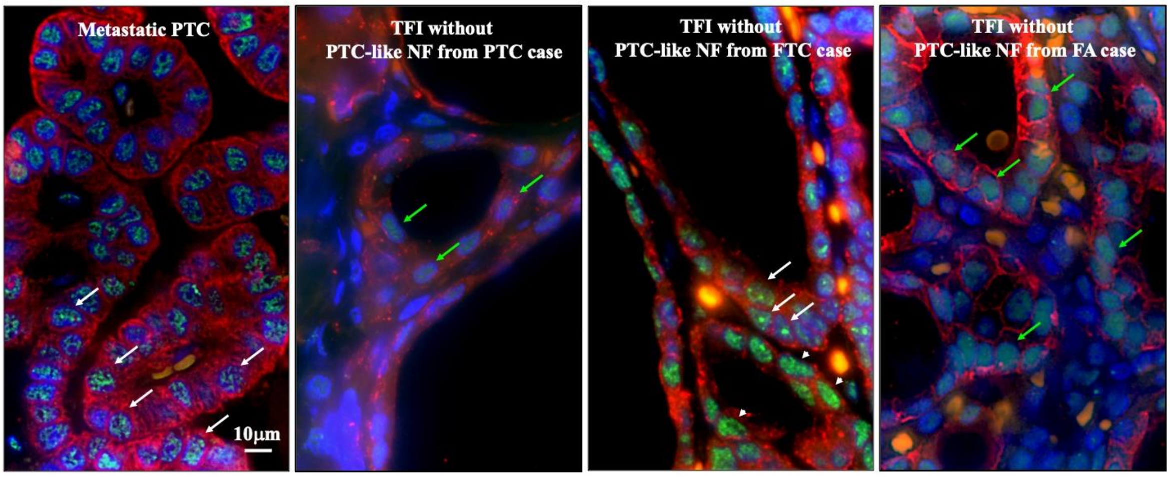

Figure S2. Immunofluorescence for 53BP1 expression in Figure 2.
